# A Combined Approach Using T2*-Weighted Dynamic Susceptibility Contrast MRI Perfusion Parameters and Radiomics to Differentiate Between Radionecrosis and Glioma Progression: A Proof-of-Concept Study

**DOI:** 10.3390/life15040606

**Published:** 2025-04-05

**Authors:** José Pablo Martínez Barbero, Francisco Javier Pérez García, David López Cornejo, Marta García Cerezo, Paula María Jiménez Gutiérrez, Luis Balderas, Miguel Lastra, Antonio Arauzo-Azofra, José M. Benítez, Antonio Jesús Láinez Ramos-Bossini

**Affiliations:** 1 Advanced Medical Imaging Group (TeCe22), Instituto de Investigación Biosanitaria de Granada (ibs.GRANADA), 18012 Granada, Spainwajaviray@gmail.com (F.J.P.G.); davidlc@correo.ugr.es (D.L.C.); martagarcia_99@outlook.com (M.G.C.); apaulajimenezg@gmail.com (P.M.J.G.); luisbalru@ugr.es (L.B.); mlastral@ugr.es (M.L.); j.m.benitez@decsai.ugr.es (J.M.B.); 2 Department of Radiology, Hospital Universitario Virgen de las Nieves, 18014 Granada, Spain; 3 Centro de Genómica e Investigación Oncológica (GENYO), 18016 Granada, Spain; 4 Department of Anesthesiology, Hospital Universitario Virgen de las Nieves, 18014 Granada, Spain; 5 Department of Computer Science and Artificial Intelligence, University of Granada, 18071 Granada, Spain; 6 Department of Software Engineering, University of Granada, 18071 Granada, Spain; 7 Department of Rural, Civil Engineering and Project Management, University of Córdoba, 14014 Córdoba, Spain; arauzo@uco.es; 8 Department of Human Anatomy and Embryology, School of Medicine, University of Granada, 18016 Granada, Spain

**Keywords:** glioma, radionecrosis, tumor progression, magnetic resonance imaging, perfusion, radiomics, machine learning

## Abstract

Differentiating tumor progression from radionecrosis in patients with treated brain glioma represents a significant clinical challenge due to overlapping imaging features. This study aimed to develop and evaluate a machine learning model that integrates radiomics features and T2*-weighted Dynamic Susceptibility Contrast MRI perfusion (DSC MRI) parameters to improve diagnostic accuracy in distinguishing these entities. A retrospective cohort of 46 patients (25 with confirmed radionecrosis, 21 with glioma progression) was analyzed. From lesion segmentation on DSC MRI, 851 radiomics features were extracted using PyRadiomics, alongside seven perfusion parameters (e.g., relative cerebral blood volume, time to peak) obtained from time–intensity curves (TICs). These features were combined into a single dataset and 14 classification algorithms were evaluated with GroupKFold cross-validation (k = 4). The top-performing model was selected based on predictive area under the curve (AUC) yield. The Logistic Regression classifier achieved the highest performance, with an AUC of 0.88, followed by multilayer perceptron and AdaBoost with AUC values of 0.85 and 0.79, respectively. The precision values were 72%, 74%, and 78% for the three models, respectively, while the accuracy was 63%, 70%, and 71%. Key predictive variables included radiomics features like wavelet-HHH_firstorder_Mean and mean normalized TIC values. Our combined approach integrating radiomics and DSC MRI parameters shows strong potential for distinguishing radionecrosis from glioma progression. However, further validation with larger cohorts is essential to confirm the generalizability of this approach.

## 1. Introduction

Glioma, the most prevalent primary malignant brain tumor in adults, represents a major clinical challenge due to its infiltrative nature and propensity for recurrence [1]. Current therapeutic strategies typically combine maximal safe surgical resection with adjuvant radiotherapy and chemotherapy, most commonly using alkylating agents such as temozolomide as part of first-line treatment [2]. One of the main problems derived from radiotherapy is the chance of developing radionecrosis (in up to 30% of patients), a radiation-induced necrotic process that mimics tumor progression on conventional magnetic resonance imaging (MRI) [3,4]. This diagnostic ambiguity carries significant clinical implications: unnecessary surgical interventions or inappropriate cessation of effective therapies may occur if radionecrosis is misclassified as progression, while delayed treatment of true progression risks worse prognosis with accelerated neurological decline [3,5].

Recent studies underscore the limitations of conventional imaging modalities in differentiating radionecrosis from recurrence/progression. For instance, contrast-enhanced T1-weighted MRI demonstrates sensitivity values as low as 31.7% for detecting progression, although high specificity values of up to 93% have been reported [4]. Advanced techniques like amino acid positron-emission tomography (PET) improve accuracy (sensitivity values 93%, specificity 100%), but limited availability and high costs restrict their routine use [4]. Perfusion-weighted MRI parameters—particularly relative cerebral blood volume (rCBV) and time to peak (TTP)—have shown promise, yet their standalone performance remains suboptimal (area under the curve [AUC], 0.75–0.88) [3,4]. The inherent heterogeneity of gliomas, compounded by treatment-induced vascular and cellular changes, necessitates multimodal approaches to overcome these diagnostic limitations [6,7].

Radiomics has emerged as a transformative tool in radiology, in general, and in neuro-oncology, in particular. Radiomics refers to the high-throughput extraction of quantitative features from medical images—such as texture patterns, intensity distributions, and morphological descriptors—that are imperceptible to visual assessment but encode subvisual heterogeneity at macroscopic and mesoscopic scales [8]. This enables the extraction of high-dimensional data from routine imaging to quantify tumor heterogeneity [3,7]. By applying computational algorithms to imaging examinations such as MR perfusion, radiomics transforms these features into mineable data, enabling the identification of phenotypic signatures correlated with tumor biology, treatment response, and clinical outcomes [9].

Recent advancements in artificial intelligence further enhance this paradigm. For instance, deep learning architectures can now achieve AUC values higher than 0.90 in many radiological challenges by synthesizing radiomics features with clinical and molecular data [3,7]. These challenges include differentiating between brain tumors, glioma grading, and glioma prognosis [10,11,12]. However, most studies to date have focused on newly diagnosed gliomas, leaving a critical gap in validated models for post-radiation scenarios, where distinguishing between radionecrosis and progression is essential for an adequate patient management [5,13].

Prior work to differentiating radionecrosis from tumor progression has predominantly focused on the analysis of either radiomics [14] or perfusion parameters in isolation [15], with variable results. However, novel strategies combining the information provided by both approaches could offer promising outcomes and would also make it possible to understand the differential significance of information based on textural (i.e., radiomics) and temporal (i.e., perfusion metrics) data sources. In fact, previous studies have applied methodological approaches combining information from radiomics and MRI perfusion data [16], but only using information from specific hemodynamic parameters as the basis to identify relevant radiomics features, precluding the analysis of the relevance of other perfusion metrics, among other limitations.

This study addresses this existing gap by developing a machine learning framework that merges radiomics features from Dynamic Susceptibility Contrast MRI perfusion (DSC MRI) with hemodynamic parameters. By integrating these complementary strategies into a single predictive model, we aim to enhance diagnostic accuracy and offer a more nuanced understanding of post-radiation lesion characterization.

## 2. Materials and Methods

### 2.1. Study Design

This study was designed and written in accordance with the Strengthening the Reporting of Observational Studies in Epidemiology (STROBE) and the Checklist for Evaluation of Radiomics Research (CLEAR, Supplementary File S1) guidelines [17,18]. We conducted a retrospective observational study of adult patients diagnosed with brain glioma (regardless of the degree) according to the WHO 2021 classification criteria [19] and treated with radiotherapy and surgical resection. On follow-up imaging, these individuals exhibited evidence of lesions suggestive of either radionecrosis or tumor progression, which were confirmed histopathologically or radiologically (follow-up >6 months). The study was conducted in the Hospital Universitario Virgen de las Nieves (Granada, Spain) and the recruitment period was from 1 January 2020 to 1 January 2024.

The eligibility criteria were as follows:

- Inclusion criteria:
  1. Histologically-confirmed brain glioma;
  2. Treatment with radiotherapy among other treatments;
  3. Lesion suspicion of recurrence or radionecrosis on follow-up DSC MRI;
  4. Minimum follow-up of 6 months.
- Exclusion criteria:
  1. Suboptimal quality of imaging examinations, including susceptibility or motion artifacts that precluded from correctly assessing the suspicious area on perfusion MRI. For quality check, several control measures were followed according to the recommendations of the American Society of Functional Neuroradiology [20];
  2. Uncertainty about the nature of the suspicious lesion due to either absence of follow-up or lack of histological confirmation.

Regarding the reference standard, pseudoprogression (i.e., radionecrosis) was defined by regression or stabilization of the suspicious lesion for at least 6 months, whilst glioma progression was defined if the suspicious lesion increased on three or more subsequent follow-up MRI studies. The study was approved by the Provincial Ethics Committee of Granada (reference, IANeuro24; approval date, 29 June 2021). The retrospective nature of the study and the minimal risk associated with reviewing anonymized imaging data led to a waiver of written informed consent. A closed-source large language model [21] was used exclusively for correcting the English writing of this manuscript.

### 2.2. Imaging Protocol and Preprocessing

All MRI studies were performed on a Philips Ingenia CX 3T system [22] with 32-channel array coils (multi-coil configuration) using a gradient-echo/echo-planar sequence. The specific imaging parameters for DSC MRI were as follows: repetition time (TR), 2059; echo time (TE), 40 ms; section thickness, 4 mm; matrix, 128 × 128; number of excitations, 1; flip angle, 75°; total acquisition time: 88.5 s; total temporal sequences: 40. All studies were de-identified to protect patient privacy, and no personally identifying information was retained. We converted the raw DICOM data to NIfTI format using dcm2niix (v. 1.0.20240202) [23] in conjunction with SimpleITK (v. 2.4.0) [24,25,26] in Python (v. 3.12.8), ensuring that original spatial parameters (e.g., orientation and pixel spacing) were preserved (Supplementary File S2).

**Figure 1:**
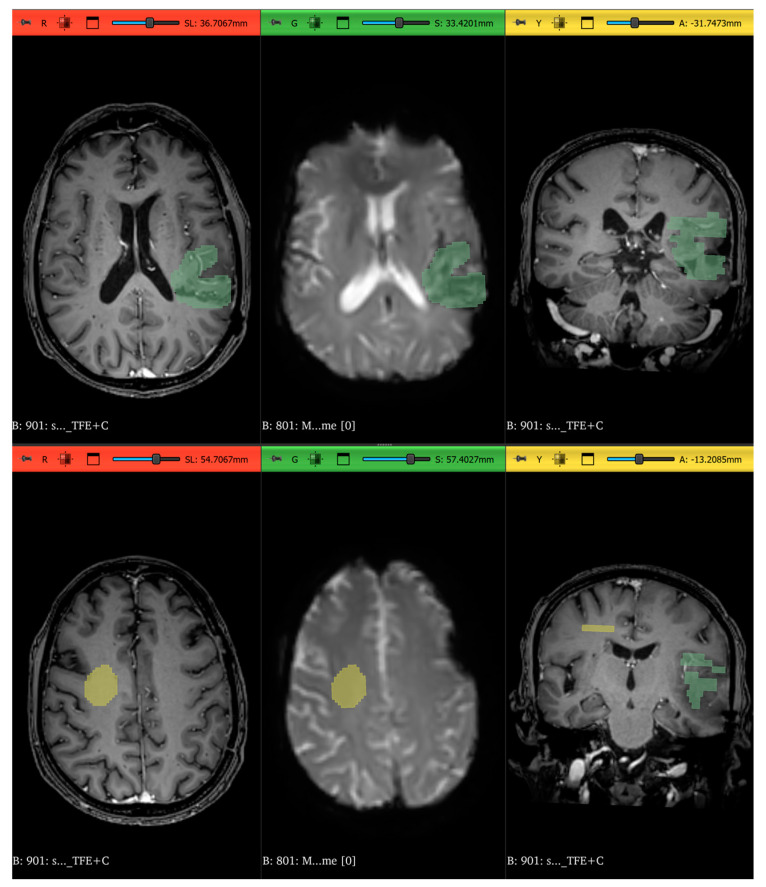
Two samples (**top** and **bottom**) of the segmentation process using 3D Slicer^®^. The segmentation of the region of interest of radionecrosis or tumor progression can be seen in green and the segmentation of the NAWM in yellow. (**Left**): axial contrast-enhanced T1-weighted image. (**Middle**): axial DSC MRI perfusion sequence. (**Right**): coronal contrast-enhanced T1-weighted image.

In 3D Slicer^®^ (v. 5.8.0), a neuroradiologist with more than 10 years of experience segmented the suspicious lesion and a region of normal-appearing white matter (NAWM) on axial DSC MRI images, following a correlative examination of contrast-enhanced T1-weighted images to ensure appropriate lesion location (Figure 1).

**Figure 2:**
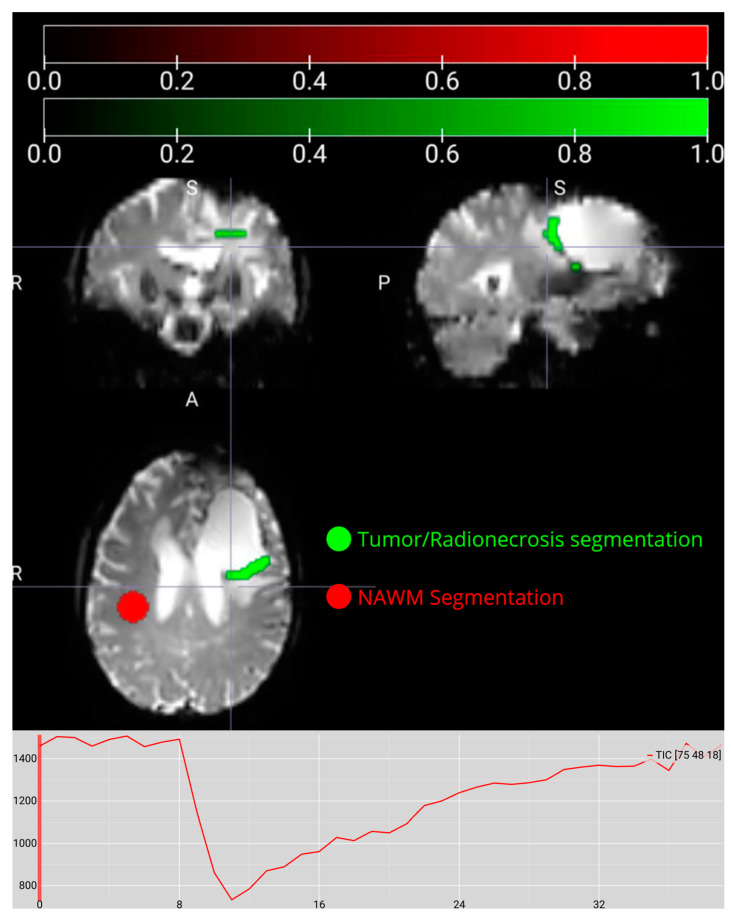
Results of the previously segmented tumor/radionecrosis and normal-appearing white matter (NAWM) in the MRIcroGL viewer, where the binary (0–1) masks for each region are displayed. The graph below shows the intensity of a voxel versus time before the preprocessing pipeline.

These steps allowed us to generate binary masks for the lesion and NAWM, which served as essential references for subsequent analyses. Once the masks were produced, we ensured that the 3D segmentations and the 4D perfusion volumes were aligned so that each timepoint in the perfusion dataset corresponded accurately to the lesion and NAWM regions using MRIcroGL (v. 1.2.20220720) [27] (Figure 2). The final output of this stage consisted of NIfTI images for aligned perfusion (4D) and binarized segmentations (3D).

### 2.3. Radiomics Features Extraction

We applied PyRadiomics (v. 3.1.0) [28] to extract 851 radiomics features from the DSC MRI volumes, focusing on both lower- and higher-order texture descriptors (Supplementary Files S3–S5). The main categories comprised first-order statistics, Gray Level Co-occurrence Matrix (GLCM), Gray Level Dependence Matrix (GLDM), Gray Level Run Length Matrix (GLRLM), Gray Level Size Zone Matrix (GLSZM), Neighborhood Gray Tone Difference Matrix (NGTDM), shape features, and wavelet-transformed variants. A detailed description of the calculations used for each of the radiomics features can be consulted in the supplementary material of the original PyRadiomics work [28]. A detailed list of all the radiomics features extracted can be consulted in Supplementary File S6.

First-order features capture fundamental statistical descriptors derived solely from the intensity distribution within the region of interest (ROI) (e.g., mean, variance, skewness, and kurtosis), without taking into account any spatial arrangement of voxels [29]. By contrast, second-order features, commonly referred to as textural parameters, examine how voxel intensities relate to one another. For instance, GLCM quantifies the frequency of specific intensity pairings at defined offsets, revealing local heterogeneity and structural patterns [30]. Together, these complementary feature sets provide a more comprehensive characterization of lesion properties by integrating both global intensity statistics and local texture information.

The extraction was performed frame by frame across each patient’s perfusion sequence. A total of 40 frames per patient were used in all cases, except for one patient’s examination, in which the last 5 frames were inadvertently lost during the transfer from the MRI machine to the PACS system and were omitted from this study. Prior to feature computation, images underwent normalization and resampling procedures set to a bin width of 5, a normalization scale of 100, and a resampled voxel spacing of [1.75, 1.75, 4]. B-spline interpolation and a padding distance of 5 mm around the ROI were also specified. Features from the lesion mask were calculated in parallel to reduce computation time, resulting in 1835 timepoint entries across all patients (46 patients with 40 time sequences plus one patient with 35 time sequences). Considering the changes in the pixels’ information of each temporal acquisition, each patient’s DSC MRI perfusion time sequence was treated as an independent observational unit for feature extraction, effectively expanding the dataset from 46 patients to 1835 unique timepoint entries.

### 2.4. Perfusion Curve Estimation and Analysis

**Figure 3:**
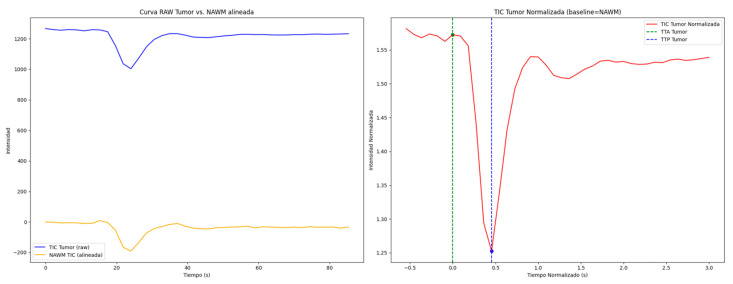
**Left graph**: Alignment of the time–intensity curves (TICs) of the lesion and normal-appearing white matter (NAWM). **Right**: TIC normalized with detection of time to arrival (TTA) and time to peak (TTP).

In addition to extracting radiomics features, we processed dynamic perfusion data using a custom Python pipeline (Supplementary Files S7 and S8). For each patient, perfusion images and their corresponding segmentation masks (tumor and NAWM) were loaded from NIfTI files, while the TR was retrieved from JSON metadata to construct a uniform temporal axis. Time–intensity curves (TICs) were then computed by averaging the signal intensities within the tumor and NAWM regions across all DSC MRI frames. The NAWM TIC established a baseline for normalizing the tumor curve, ensuring a pre-contrast value of one, and the normalized tumor TIC was temporally aligned by shifting its time-to-arrival (TTA) to zero. The TTP was identified as the minimum point of the normalized tumor curve, and several perfusion parameters were subsequently calculated—including the rCBV via trapezoidal integration, percentage signal recovery (PSR), mean transit time (MTT), maximal falling slope, and the maximal signal intensity difference (MSID) for both the tumor and NAWM. Finally, dual-panel figures contrasting the raw and normalized TICs were generated (Figure 3).

After extracting both radiomics features and perfusion parameters, we merged these datasets into a single one that associated each timepoint with a patient identifier, the corresponding label (progression or radionecrosis as 0 and 1), and relevant curve-derived parameters. Non-informative columns—such as those providing software version numbers—were removed to avoid bias. This preprocessed and cleaned dataset was stored in a spreadsheet containing the perfusion parameters and radiomics features.

### 2.5. Machine Learning Pipeline and Statistical Analysis

We used the scikit-learn (v. 1.6) [31] classification module to compare 14 classification algorithms (AdaBoost, CatBoost, DecisionTree, ExtraTrees, GaussianNB, GradientBoosting, KNeighbors, LDA, LightGBM, Logistic Regression, multilayer perceptron [MLP], RandomForest, SVC, and XGBoost) (Supplementary File S9). The goal was to determine which approach performed best in classifying radionecrosis versus tumor progression (i.e., dependent variable). Since each patient contributed multiple timepoints, we employed GroupKFold cross-validation with k = 4 to ensure that timepoints from the same patient did not appear in differents folds and were randomly distributed. The random distribution generated by the seed of this experiment can be consulted in Supplementary File S10.

For the evaluation of each method, we generated AUC plots for each iteration applied to timepoints of the cross-validation and the mean of these, as well as feature importance plots for each model, performance metrics, and confusion matrices.

## 3. Results

### 3.1. Characteristics of Patients and Imaging Data

**Figure 4:**
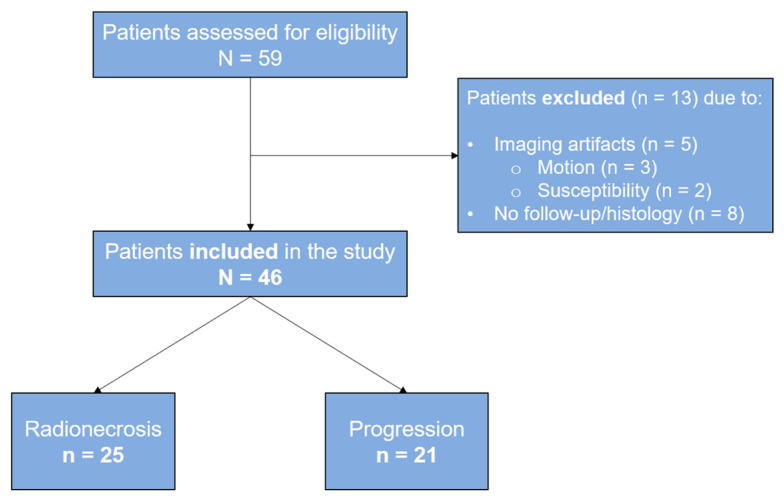
Flow diagram of the patients included in the study.

We included a total of 46 patients with completely resected brain gliomas, similarly distributed between radionecrosis (*n* = 25) and progression (*n* = 21). Figure 4 shows the flow diagram of the study.

The mean age was 54.4 years, and 24 (52.2%) patients were women. Table 1 describes the main characteristics of the sample analyzed in this study. As previously described, this cohort generated 1835 timepoint entries, each containing 851 radiomics features and seven perfusion parameters extracted from TICs.

### 3.2. Performance of the Selected Models

**Figure 5:**
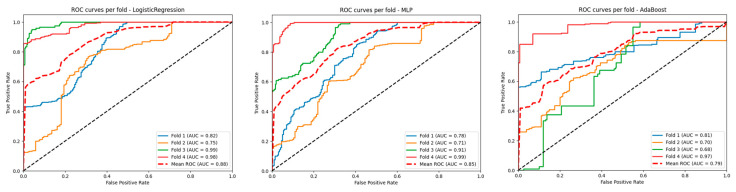
Receiver Operating Characteristic (ROC) curve analysis for the Logistic Regression (**left**), multilayer perceptron (MLP) (**middle**), and AdaBoost (**right**) models; 0, radionecrosis; 1, progression.

**Figure 6:**
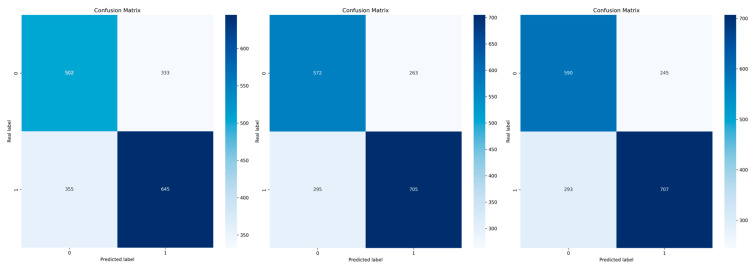
Confusion matrices of the progression and radionecrosis categories indicating true and predicted observations for Logistic Regression (**left**), multilayer perceptron (MLP) (**middle**), and AdaBoost (**right**). The total number of categories corresponds to the total number of patients in the training set multiplied by the number of temporal DSC MRI sequences.

**Figure 7:**
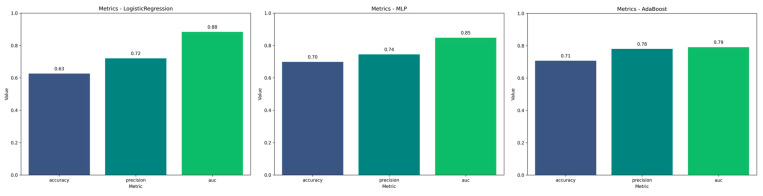
Accuracy results of the Logistic Regression (**left**), multilayer perceptron (MLP) (**middle**), and AdaBoost (**right**) classification models.

Fourteen models were assessed in our evaluation process. The best-performing classifier was Logistic Regression, which achieved an AUC of 0.88 (Figure 5) under GroupKFold cross-validation, followed by MLP and AdaBoost with AUC values of 0.85 and 0.79, respectively. The confusion matrices to visualize the results of the cross-validation in training for the three models are shown in Figure 6. The precision values for the three models were 72%, 74%, and 78%, respectively, while the accuracy was 63%, 70%, and 71%, respectively (Figure 7). The main contributors to the models that allow the assessment of individual variables (Logistic Regression and AdaBoost) were perfusion-based features, in particular MTT, MSID, TTA, max slope, PSR, and rCBV, together with radiometric features such as wavelet-HHH_firstorder_mean or wavelet-HHH_firstorder_skewness. A summary of the performance of the other models can be found in Table 2.

### 3.3. Feature Importance

**Figure 8:**
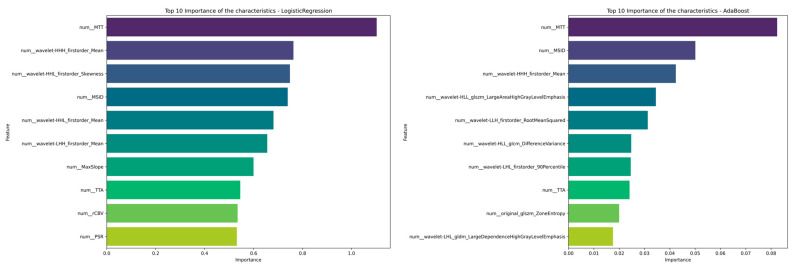
Significance plots of the variables included in the Logistic Regression (**left**) and AdaBoost (**right**) classifiers.

Analysis of feature relevance confirmed that perfusion-derived parameters played a pivotal role (Figure 8). Among radiomics features, wavelet-HHH_firstorder_Mean was the most important predictor. This feature represents the mean intensity of voxels in an image processed with a wavelet-HHH filter, which enhances high-frequency structures in all directions. Several other wavelet-based features demonstrated significant discriminative power like wavelet-HHL_firstorder_Skewness, wavelet-HHL_firstorder_Mean, wavelet-LHH_firstorder_Mean, wavelet-HLL_glszm_LargeAreaHighGrayLevelEmphasis, and wavelet-HLL_glsz_LargeAreaHighGrayLevelEmphasis.

Conventional perfusion parameters also showed significant predictive value. In the Logistic Regression classifier, six out of the seven evaluated parameters were among the ten most important variables of the top-performing models, while three were included in the AdaBoost classifier.

## 4. Discussion

In this study, we developed an integrated machine learning framework that combined radiomics features extracted from DSC MRI with several perfusion parameters to differentiate tumor progression from radionecrosis in glioma patients. Our best-performing classifier, Logistic Regression, achieved an outstanding AUC of 0.88 under GroupKFold cross-validation. These findings underscore the potential of multimodal data fusion to surpass the performance reported in previous studies that have evaluated perfusion parameters or radiomics in isolation.

Our results demonstrate that classical perfusion metrics—including rCBV, CBF, MTT, mean TIC, and MSID—were among the top contributors in classifying tumor progression. This is in concordance with earlier works indicating that recurrent tumors typically exhibit elevated rCBV and altered hemodynamics relative to radionecrosis [32,33]. Increased rCBV and CBF values are strongly associated with tumor recurrence, whereas radionecrosis tends to present with lower perfusion indices, likely due to radiation-induced vascular damage and fibrosis [34,35,36,37]. Similarly, our observation that the mean normalized TIC and rCBV significantly predict progression aligns with these studies and reinforces the clinical relevance of DSC MRI-derived perfusion measures.

Furthermore, our radiomics analysis revealed that wavelet-transformed features provided additional discriminative power. In our model, wavelet-HHH_firstorder_Mean was inversely associated with progression, suggesting that certain intensity patterns in these frequency bands may reflect the aggressive biology of recurrent gliomas. Prior works highlighted that wavelet-based texture features are capable of capturing intratumoral heterogeneity and have been correlated with molecular signatures in glioblastoma [38]. These results support our finding that incorporating wavelet-based radiomics features, which capture subtle textural differences beyond what is apparent in the raw perfusion data, enhances model performance. These areas tend to exhibit a more homogeneous and fibrotic structure with altered signal recovery characteristics, as suggested by recent studies using texture analysis to differentiate post-radiation changes from recurrent tumors [39,40,41]. Although the literature on radiomics correlates of radionecrosis is not as extensive as that for tumor progression, our findings are consistent with the hypothesis that radionecrosis presents a unique textural signature on DSC MRI.

The combination of radiomics and MRI perfusion parameters in the study of glioma is not completely new. Previous studies have tackled a set of diagnostic and prognostic problems using this combined approach. For instance, Crisi et al. [42] evaluated 92 quantitative features on rCBV and rCBF maps to predict the MGMT promoter methylation status on brain gliomas. They found that the best-performing model (MLP) using the five most relevant imaging features showed good performance, with an AUC of 0.84. A similar approach to predict glioma grade was followed by Hashido et al. [43], who found that a radiomics model combined with rCBV from DSC MRI achieved excellent performance (AUC = 0.962) in differentiating between low- and high-grade gliomas. Interestingly, they also found that a similar model based on arterial spin labeling perfusion MRI instead of DSC MRI showed non-significantly lower results. In addition, Sudre et al. [44] applied a random forest model to combined radiomics–DSC MRI metrics, achieving good results to predict the IDH mutation status and glioma grade (WHO II–IV), with overall specificity of 77% and sensitivity of 65%. Notably, the results for WHO glioma grade classification were significantly worse (53% of gliomas were correctly classified).

To our knowledge, only two previous studies have explored a combined approach based on radiomics and perfusion MRI to differentiate between radionecrosis and glioma progression. Elshafeey et al. [16] aimed to differentiate between pseudoprogression and true progression in a cohort of 98 patients with glioblastoma. They extracted over 300 radiomics features from rCBV and kTrans perfusion maps and applied a support vector machine model, achieving outstanding results with AUC values of 0.94 and 0.90, respectively. Similarly, Kim et al. approached the same problem in a cohort of 61 patients with glioblastoma [45]. They developed a combined approach including radiomics and multiparametric (contrast-enhanced T1, FLAIR, diffusion, and rCBV map parameters) MRI, achieving an AUC of 0.90. Our approach is different to these two previous studies, since we merged the information provided by the TIC-derived parameters with that from radiomics features extracted in the ROI (i.e., as independent variables). Conversely, the previous authors extracted radiomics features from maps of specific perfusion parameters (e.g., rCBV).

The main advantages of our conceptual approach lie in the combination of TIC-derived parameters and radiomics features, providing cues regarding the relative contribution of both approaches in each machine learning method, facilitating their explicability and opening interesting questions to be approached in future studies. Notably, while DSC MRI-derived parameters provide physiologic insights regarding vascular perfusion and hemodynamics, radiomics features capture underlying tissue heterogeneity. In our study, the 10 most relevant variables of the Logistic Regression model included more TIC-derived variables, leading to an AUC of 0.88 and precision of 78%, whereas AdaBoost included more radiomics features than TIC-derived variables, leading to an AUC of 0.79 and the same precision (78%).

Nevertheless, some relative discrepancies exist between our findings and earlier reports that have evaluated individual DSC MRI parameters in isolation. For example, some studies have noted that the standalone performance of perfusion parameters such as TTP is modest [46], which may be attributable to inter-patient variability in acquisition protocols or postprocessing methods. Our study suggests that combining these traditional parameters with radiomic texture features mitigates such variability and enhances the robustness of the predictive model.

Several limitations must be acknowledged. First, our cohort was small (46 patients) due to limited availability of eligible cases in our institution. However, the sample size obtained is comparable to that of previous works on the topic [41,44], and the data distribution and statistical results in the present study, along with the balance of the outcome categories, support the validity of our conclusions—especially considering the conceptual nature of our approach. Similarly, all MRI examinations were performed in the same scanner, which also limits the external validity of our findings. In addition, the gold standard (histopathology or radiological follow-up >6 months) does not totally exclude selection bias, e.g., delayed recurrence misclassified as radionecrosis. Moreover, one MRI study had 35 temporal frames rather than the 40 used for most subjects due to a data transfer error between the MRI scanner and the PACS, potentially introducing variability in the extracted radiomics features. However, the missing time frames were at the end of the time series; thus, it is unlikely that they significantly affected the TIC-derived parameters.

Another important limitation lies in the high dimensionality of the radiomics data which, combined with a relatively limited sample size and lack of explicit feature selection, poses a risk for model overfitting. In addition, the proposed model is a classifier that uses temporal sequences as observational units instead of patients. We have observed that it normally classifies all 40 temporal sequences of the same patient in the same way, but when this is not the case (e.g., in future studies), a threshold-based system should be generated to determine whether the patient belongs to one class or another. Finally, the models’ accuracy was not compared with the performance of neuroradiologists. Of note, such a comparison is difficult and sometimes unrealistic, since the degree of uncertainty of the neuroradiologist is high in many cases, which prevents them from giving a definitive diagnosis and makes them suggest close imaging follow-up. The RANO criteria would be an appropriate benchmark in future studies, although they are usually restricted to clinical trials and not commonly used in real-world practice [47].

Future research should overcome these limitations and explore other approaches that were out of the scope of the present study. For instance, whilst we focused on the radiomics features contained in PyRadiomics, other texture feature analysis tools such as MaZda [48] could offer additional information of interest. Similarly, although we applied all the state-of-the-art classifiers, including neural networks (MLP), more complex architectures like deep learning models were not used due to the small size of the dataset and the unavailability of a pre-trained model for similar cases. Future studies should explore the potential of these models. In sum, external validation in larger, multicenter cohorts will be necessary to confirm the robustness and utility of our integrated machine learning approach.

## 5. Conclusions

We demonstrated that a radiomic–perfusion approach—combining DSC MRI perfusion parameters with texture-based features—can reliably distinguish radionecrosis from tumor progression in a limited glioma patient cohort. Our top-performing model achieved an AUC of 0.88, underscoring the promise of integrating conventional perfusion measures with higher-order radiomic descriptors. However, the small sample size limits the generalization of these findings; thus, these results should be viewed as an initial step toward more comprehensive investigations.

## Figures and Tables

**Table 1:** Characteristics of the patients and gliomas analyzed in the study. * Three cases located in the cerebellum (one in each hemisphere and another one in the vermis), and one in the basal ganglia.

	Total (N = 46)	Radionecrosis (n = 25)	Progression (n = 21)	*p*-Value
Women	24 (52.2)	11 (44)	13 (61.9)	0.226
Age	54.4 ± 11.5	56.9 ± 11.7	51.5 ± 10.8	0.110
Location				
Frontal	18 (39.1)	9 (36)	9 (42.9)	
Parietal	6 (13.0)	5 (20)	1 (4.8)	
Temporal	16 (34.8)	6 (24)	10 (47.6)	0.173
Occipital	2 (4.3)	2 (8)	0 (0)	
Other *	4 (8.7)	3 (12)	1 (4.8)	
Side = right	18 (39.1)	11 (44)	7 (33.3)	0.551
High-grade glioma	36 (78.2)	18 (72)	18 (85.7)	0.306

**Table 2:** Performance metrics of the fourteen machine learning models assessed. AUC, area under the curve. MLP, multilayer perceptron. SVC, support vector classifier. kNN, K-Nearest Neighbors. LDA, Linear Discriminant Analysis. LightGBM, Light Gradient-Boosting Machine. XGBoost, eXtreme Gradient Boosting.

Model	Accuracy	Precision	AUC mean
LogisticRegression	0.6264	0.7205	0.8842
MLP	0.6986	0.7449	0.8479
AdaBoost	0.7074	0.7803	0.7915
SVC	0.6727	0.7074	0.7946
GradientBoosting	0.6899	0.6968	0.7854
kNN	0.7127	0.7327	0.7388
CatBoost	0.5673	0.6388	0.7326
ExtraTrees	0.6041	0.7038	0.7185
LDA	0.6326	0.7532	0.7055
LightGBM	0.6015	0.6812	0.6993
DecisionTree	0.5906	0.6568	0.6279
GaussianNB	0.5236	0.6562	0.5944
XGBoost	0.6367	0.6854	0.7420
RandomForest	0.5695	0.6591	0.6781

## Data Availability

The imaging data presented in this study are available on request from the corresponding authors due to restrictions imposed by the Ethics Committee which approved the study protocol. The provided scripts and related information are publicly available on GitHub (URL: https://github.com/dlcornejo/DSC-MRI-and-radiomics-to-differentiate-between-radionecrosis-and-glioma-progression, accessed on 25 March 2025).

## Supplementary Materials

The following supporting information can be downloaded at https://www.mdpi.com/article/10.3390/life15040606/s1: Supplementary File S1 (Checklist.pdf): Checklist for evaluation of radiomics research. Supplementary File S2 (1.py): Script that converts medical imaging files (DICOM, NRRD) to NIfTI, aligns perfusion with segmentation, and extracts imaging metrics for patients. Supplementary File S3 (2.py): Script that extracts radiomics features from perfusion images using PyRadiomics, processing multiple patients in parallel. Supplementary File S4 (3.py): Script that reads the extracted features and counts the number of timepoints per patient. Supplementary File S5 (4.py): Script that cleans the extracted features by removing unnecessary columns and saves the cleaned data. Supplementary File S6 (RadiomicsFeatures.xlsx): List of radiomics features extracted with PyRadiomics. Supplementary File S7 (5.py): Script that computes time–intensity curves (TICs) from perfusion images, normalizing and smoothing them for analysis. Supplementary File S8 (6.py): Script that merges extracted features with normalized TICs into a single dataset. Supplementary File S9 (7.py): Script that uses scikit-learn to train machine learning models for progression prediction using extracted features. Supplementary File S10 (PatientFolds.pdf): Distribution of patients in the different folds in this experiment.

## Abbreviations

The following abbreviations are used in this manuscript:

AUC	Area under the curve
DSC	Dynamic Susceptibility Contrast
GLCM	Gray Level Co-occurrence Matrix
GLDM	Gray Level Dependence Matrix
GLRLM	Gray Level Run Length Matrix
GLSZM	Gray Level Size Zone Matrix
MRI	Magnetic resonance imaging
NAWM	Normal-appearing white matter
NGTDM	Neighborhood Gray Tone Difference Matrix
PSR	Percentage signal recovery
rCBV	Relative cerebral blood volume
ROI	Region of interest
TIC	Time–intensity curve
TTA	Time to arrival
TTP	Time to peak
TR	Repetition time
