# Supplementary material for: A Combined Approach Using T2*-Weighted Dynamic Susceptibility Contrast MRI Perfusion Parameters and Radiomics to Differentiate Between Radionecrosis and Glioma Progression: A Proof-of-Concept Study
Source: Life (Basel). 2025 Apr 5;15(4):606. doi: 10.3390/life15040606 (PMC12028526; doi:10.3390/life15040606)
Supplement: Supplementary file 1 [file life-15-00606-s001.zip › Checklist.pdf]

CLEAR Checklist v1.0

Note: Use the checklist in conjunction with the main text for clarification of all items.
Yes, details provided; No, details not provided; n/e, not essential; n/a, not applicable; Page, page number

Section	No.	Item	Yes	No	n/a	Page
**Title**						
	1	Relevant title, specifying the radiomic methodology	☑	☐	☐	1
**Abstract**						
	2	Structured summary with relevant information	☑	☐	☐	1
**Keywords**						
	3	Relevant keywords for radiomics	☑	☐	☐	1
**Introduction**						
	4	Scientific or clinical background	☑	☐	☐	1-2
	5	Rationale for using a radiomic approach	☑	☐	☐	2
	6	Study objective(s)	☑	☐	☐	2
**Method**						
Study design	7	Adherence to guidelines or checklists (e.g., CLEAR checklist)	☑	☐	☐	11
	8	Ethical details (e.g., approval, consent, data protection)	☑	☐	☐	3
	9	Sample size calculation	☐	☐	☑	
	10	Study nature (e.g., retrospective, prospective)	☑	☐	☐	3
	11	Eligibility criteria	☑	☐	☐	3
	12	Flowchart for technical pipeline	☐	☑	☐	
Data	13	Data source (e.g., private, public)	☑	☐	☐	3
	14	Data overlap	☑	☐	☐	3
	15	Data split methodology	☑	☐	☐	6
	16	Imaging protocol (i.e., image acquisition and processing)	☐	☑	☐	
	17	Definition of non-radiomic predictor variables	☑	☐	☐	2
	18	Definition of the reference standard (i.e., outcome variable)	☐	☑	☐	
Segmentation	19	Segmentation strategy	☑	☐	☐	2
	20	Details of operators performing segmentation	☑	☐	☐	2
Pre-processing	21	Image pre-processing details	☑	☐	☐	2-6

Section	No.	Item	Yes	No	n/a	Page
	22	Resampling method and its parameters	✓			5
	23	Discretization method and its parameters	✓			5
	24	Image types (e.g., original, filtered, transformed)	✓			6
Feature extraction	25	Feature extraction method	✓			5
	26	Feature classes	✓			5
	27	Number of features	✓			5
	28	Default configuration statement for remaining parameters	✓			5
Data preparation	29	Handling of missing data	✓			5
	30	Details of class imbalance	✓			6
	31	Details of segmentation reliability analysis			✓	
	32	Feature scaling details (e.g., normalization, standardization)	✓			5
	33	Dimension reduction details	✓			5
Modeling	34	Algorithm details	✓			6
	35	Training and tuning details	✓			6
	36	Handling of confounders	✓			6
	37	Model selection strategy	✓			7-9
Evaluation	38	Testing technique (e.g., internal, external)	✓			7-9
	39	Performance metrics and rationale for choosing	✓			7-9
	40	Uncertainty evaluation and measures (e.g., confidence intervals)	✓			7-9
	41	Statistical performance comparison (e.g., DeLong's test)			✓	
	42	Comparison with non-radiomic and combined methods	✓			9-10
	43	Interpretability and explainability methods	✓			9-10
**Results**						
	44	Baseline demographic and clinical characteristics		✓		
	45	Flowchart for eligibility criteria		✓		
	46	Feature statistics (e.g., reproducibility, feature selection)	✓			9
	47	Model performance evaluation	✓			7-9
	48	Comparison with non-radiomic and combined approaches	✓			9-10
**Discussion**						

Section	No.	Item	Yes	No	n/a	Page
	49	Overview of important findings	☑	☐	☐	9-11
	50	Previous works with differences from the current study	☑	☐	☐	9-11
	51	Practical implications	☑	☐	☐	11
	52	Strengths and limitations (e.g., bias and generalizability issues)	☑	☐	☐	9-11
**Open Science**						
Data availability	53	Sharing images along with segmentation data [n/e]	☑	☐	☐	11
	54	Sharing radiomic feature data	☑	☐	☐	11
Code availability	55	Sharing pre-processing scripts or settings	☑	☐	☐	11
	56	Sharing source code for modeling	☑	☐	☐	11
Model availability	57	Sharing final model files	☑	☐	☐	11
	58	Sharing a ready-to-use system [n/e]	☐	☑	☐	

Kocak B, Baessler B, Bakas S, Cuocolo R, Fedorov A, Maier-Hein L, Mercaldo N, Müller H, Orlhac F, Pinto Dos Santos D, Stanzione A, Ugga L, Zwanenburg A. CheckList for EvaluAtion of Radiomics research (CLEAR): a step-by-step reporting guideline for authors and reviewers endorsed by ESR and EuSoMII. Insights Imaging. 2023 May 4;14(1):75. doi: 10.1186/s13244-023-01415-8
