# Supplementary material for: A Combined Approach Using T2*-Weighted Dynamic Susceptibility Contrast MRI Perfusion Parameters and Radiomics to Differentiate Between Radionecrosis and Glioma Progression: A Proof-of-Concept Study
Source: Life (Basel). 2025 Apr 5;15(4):606. doi: 10.3390/life15040606 (PMC12028526; doi:10.3390/life15040606)
Supplement: Supplementary file 1 [file life-15-00606-s001.zip › PatientFolds.pdf]

Supplementary file 2: Distribution of patients in the different folds in this experiment.

Fold 1	Fold 2	Fold 3	Fold 4
'Patient_1'	'Patient_2'	'Patient_7'	'Patient_3'
'Patient_5'	'Patient_10'	'Patient_13'	'Patient_4'
'Patient_8'	'Patient_14'	'Patient_17'	'Patient_6'
'Patient_9'	'Patient_18'	'Patient_20'	'Patient_12'
'Patient_11'	'Patient_21'	'Patient_24'	'Patient_16'
'Patient_15'	'Patient_25'	'Patient_28'	'Patient_19'
'Patient_22'	'Patient_31'	'Patient_29'	'Patient_23'
'Patient_26'	'Patient_34'	'Patient_33'	'Patient_27'
'Patient_30'	'Patient_38'	'Patient_37'	'Patient_32'
'Patient_35'	'Patient_41'	'Patient_40'	'Patient_36'
'Patient_39'	'Patient_42'	'Patient_45'	'Patient_44'
'Patient_43'	'Patient_46'		
